# When Teratomas Turn Brutal: From Indolent to Invasive in Stage IIIB Ovarian Squamous Cell Carcinoma

**DOI:** 10.7759/cureus.88090

**Published:** 2025-07-16

**Authors:** Nasim Salimiaghdam, Ahmed Mustafa, Afshin Hamidi, Alsu Ramazanova, Emily Chen

**Affiliations:** 1 Internal Medicine Residency Program, Capital Health Regional Medical Center, Trenton, USA; 2 Internal Medicine, Capital Health Regional Medical Center, Trenton, USA; 3 Endocrinology and Diabetes, Arrowhead Regional Medical Center, Colton, USA; 4 Hematology and Medical Oncology, Capital Health Regional Medical Center, Trenton, USA

**Keywords:** adnexal mass, invasive, mature teratoma, ovarian cancer, squamous cell carcinoma

## Abstract

This case report identifies a 74-year-old female with Stage IIIB ovarian cancer, with a specific diagnosis of squamous cell carcinoma arising in a mature teratoma. She sustained surgical cytoreduction that included gynecologic oncology, general surgery, and urology. Post-operative care was coordinated to deal with early complications and recovery by working together as a multidisciplinary team. The case delves into the rare pathophysiology of malignant transformation in teratoma related to ovarian cancer, the principles of staging, and current treatment methods. This case further emphasizes the prognostic importance of mesoappendiceal involvement, a factor that independently contributes to upstaging and indicates a higher risk for recurrence. Follow-up care consisted of advanced imaging, options for adjuvant chemotherapy, and genetic counseling/referral for testing. This case report recognizes that early identification, the importance of multidisciplinary care, and follow-up based on rare ovarian malignancies are found in each of these issues.

## Introduction

Ovarian cancer stands out as one of the top causes of cancer-related deaths among women around the globe. Unfortunately, it is often diagnosed at later stages because the early signs are often silent. While most ovarian cancers start from epithelial cells, there are several other types, including germ-cell tumors. Among these, teratomas - benign tumors that develop from the three embryonic germ layers - are the most frequently seen germ cell tumors in the ovaries [1].

Typically, mature teratomas are benign, but in rare instances, they can turn malignant [2]. Malignant transformation occurs in approximately 1%-2% of all mature cystic teratomas, and when it does, squamous cell carcinoma (SCC) is the most common histologic type, accounting for less than 2% of all ovarian malignancies overall [3]. These tumors are commonly found incidentally, but can also present as abdominal pain or a palpable mass, particularly in post-menopausal women. Imaging modalities (CT and MRI) are integral to preoperative characterization. A definitive diagnosis occurs with surgical resection and histologic analysis - hematoxylin and eosin (H&E) stain. This case illustrates the diagnosis and multidisciplinary management of an unusual ovarian SCC arising from a mature teratoma, with a focus on imaging, surgical, and pathological correlation [4]. Recently published literature suggested mechanisms such as mutations of the p53 tumor suppressor, persistent human papillomavirus (HPV) infection, and proinflammatory signaling pathways as possible contributors to this malignant transformation [5,6]. These studies suggest that mature teratomas may undergo a process nearly carcinogenic in nature and must take sequential and stepwise genetic and microenvironmental pathways toward SCC, while hard evidence is still scant because of the rarity of such disease. While squamous transformation is rare, the aggressive nature of the tumor and the late detection of the disease stage mean this development is clinically significant. In this context, our case describes the diagnostic and therapeutic dilemmas that we encountered, based on the experience of managing a 74-year-old woman with a Stage IIIB presentation overall. The aggressive nature of SCC makes it difficult to manage clinically, usually needing a multi-modal approach combining WS, chemotherapy, and, in some cases, radiation therapy. Due to the rarity of these transformations, each case is typically unique, making diagnosis, staging, and treatment difficult [7,8].

A 74-year-old woman was diagnosed with Stage IIIB ovarian cancer due to SCC from a mature teratoma. She has advanced disease involving the mesoappendiceal and pelvic areas, requiring radical surgical intervention. After surgical resection, her treatment includes chemotherapy and ongoing follow-up for monitoring and genetic testing.

## Case presentation

A 74-year-old woman with a history of hypertension, hyperlipidemia, hypothyroidism, and osteoarthritis presented with progressive abdominal symptoms over three months. The symptoms began as vague abdominal discomfort, which developed into early satiety, a feeling of pelvic pressure, urinary frequency, and 10 pounds of unintentional weight loss. The change in appetite was more apparent during the last month before presentation.

Her past surgical history included laparoscopic cholecystectomy and cesarean section. There was no known family history of gynecologic or colorectal malignancy. For social history, she was functionally independent and lived alone, but her daughter would assist her intermittently.

Initial evaluation

On examination, she appeared alert and oriented, with stable vital signs. Her abdomen was distended with a mildly tender lower abdomen, and upon palpation, there was a mass that extended from the pelvis to the umbilicus. The pelvic examination was most concerning for a large, fixed adnexal mass, which was only able to move freely in the left-right axis.

Preoperative workup

**Figure 1:**
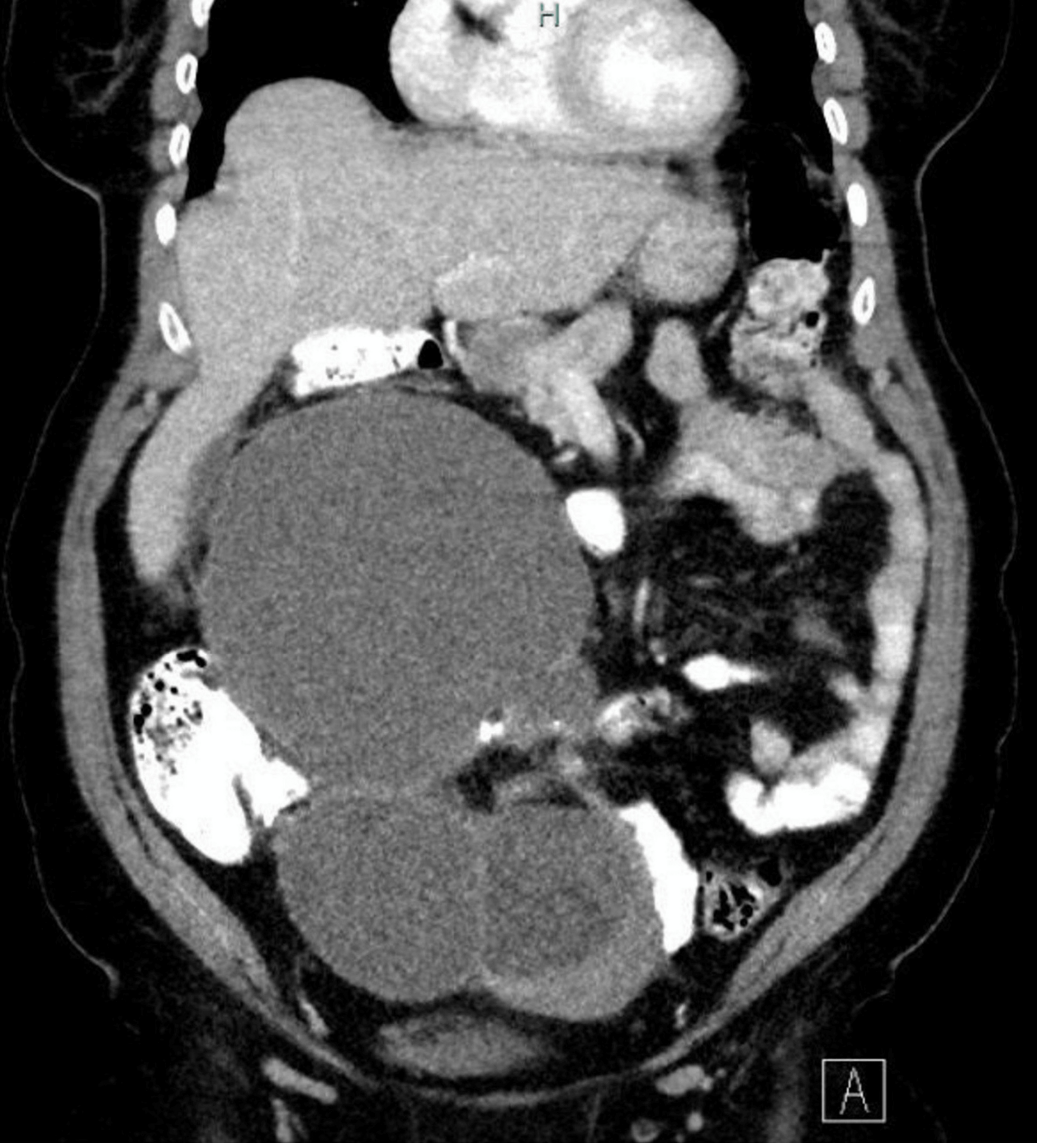
Contrasted axial CT imaging of the abdomen and pelvis Contrasted axial CT imaging of the abdomen and pelvis reveals a large (27 × 13 × 8.5 cm), well-circumscribed, heterogeneous pelvic mass consisting of solid and cystic portions, consistent with a mature teratoma with some degree of malignant transformation. The mass is displacing adjacent bowel loops and compressing the urinary bladder inferiorly. There is an associated soft tissue extension involving the mesoappendix, concerning for local invasion. The liver, kidneys, and upper abdomen are seen and appear uninvolved at current levels.

There was laboratory evidence of normocytic anemia (Hgb 10.4 g/dL), elevated CA-125 (127 U/mL), and mildly elevated ESR and CRP in laboratory testing. Liver and kidney function panels were normal. Transvaginal and abdominal ultrasound showed a complex, lobulated solid-cystic mass in the left adnexa measuring about 18 × 10 cm. CT abdomen/pelvis with IV contrast demonstrated a large, heterogeneous pelvic mass measuring 27 × 13 × 8.5 cm, consistent with a mature cystic teratoma of the left ovary (Figure 1). The tumor showed extensive keratinization as well as keratin pearls. Further results showed nodular thickening of the mesoappendix and omental stranding with no evidence of distant metastasis, and a 4 mm lingular nodule was seen on chest CT, which was indeterminate and scheduled for surveillance imaging.

**Figure 2:**
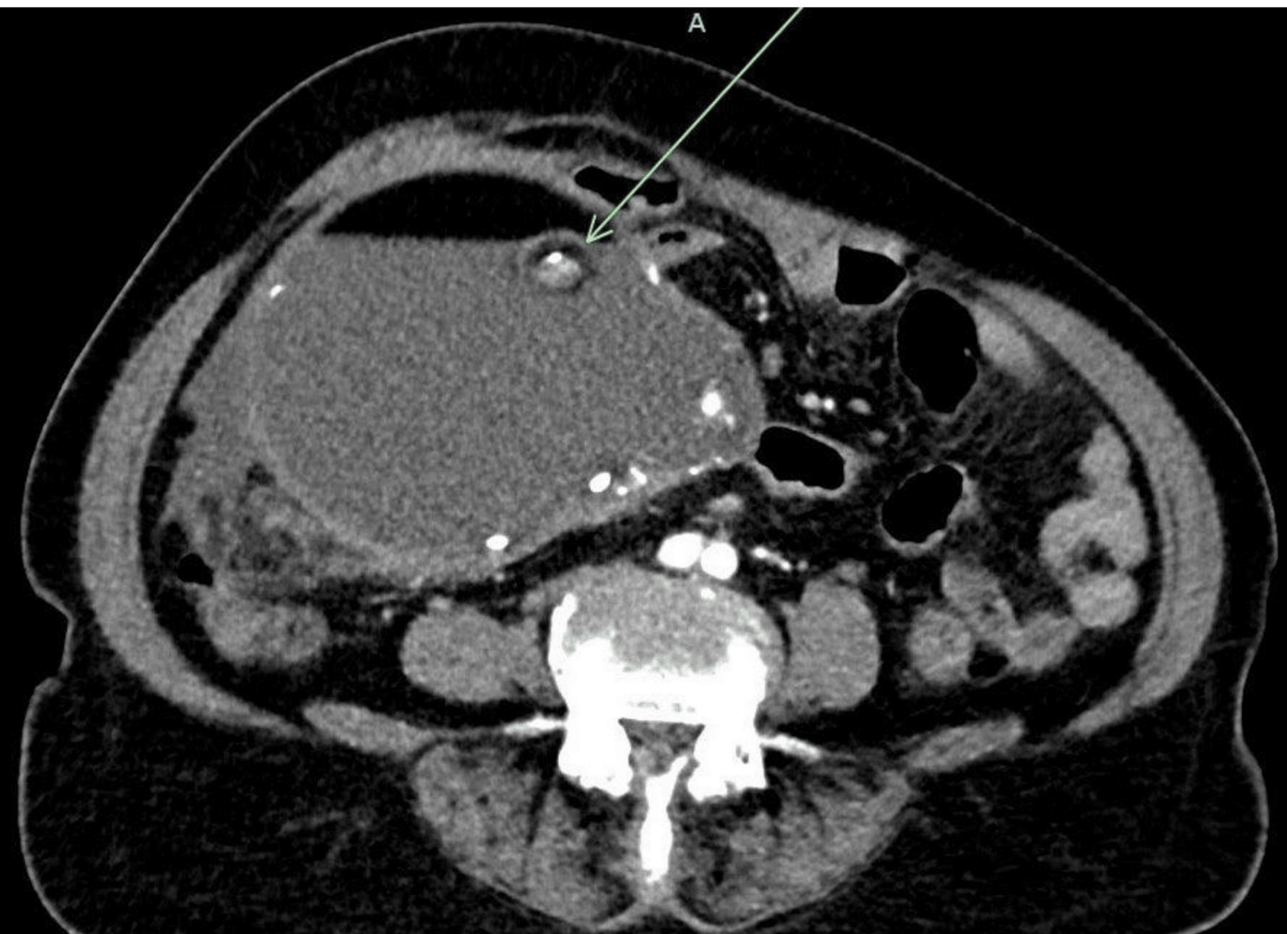
Axial contrast-enhanced CT image of the pelvis Axial contrast-enhanced CT image of the pelvis demonstrating a large, multiloculated cystic mass arising from the right adnexa, consistent with an ovarian teratoma with malignant transformation. The lesion demonstrates jagged internal density with areas of focal soft tissue enhancement. The green arrow delineates an area of capsular disruption, or rupture into the tumor, which was one of the reasons for assigning it FIGO (International Federation of Gynecology and Obstetrics) Stage IIIB (due to capsular disruption and mesoappendiceal involvement). The bowel loops and bladder are displaced due to mass effect; there are no gross ascites or peritoneal implants noted at this level.

The contrast-enhanced abdominal CT demonstrated a complex pelvic mass with solid and cystic components, irregular borders, and suspected invasion, indicating malignant transformation of a mature teratoma and possible SCC (Figure 2).

**Figure 3:**
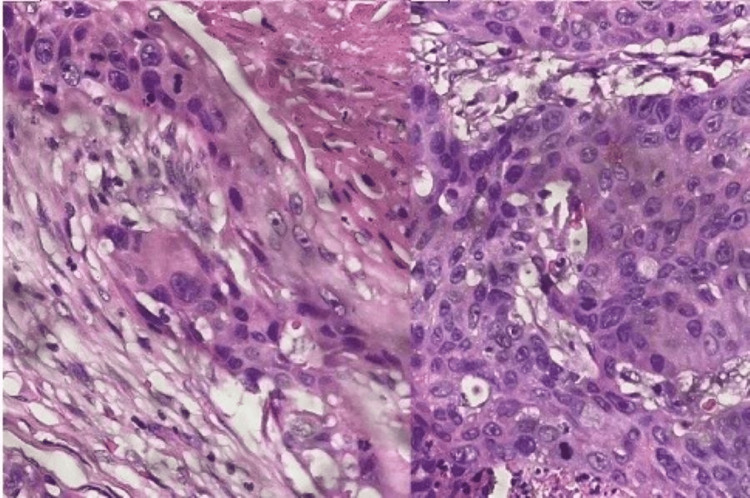
Histopathologic section (H&E stain, original magnification x200) Histopathologic section (H&E stain, original magnification ×200) showing invasive squamous cell carcinoma within a mature cystic teratoma of the ovary. The image shows keratin pearls (concentric eosinophilic structures with keratinized squamous cells) and intercellular bridges (thin cytoplasmic connections between tumor cells), which represent classic characteristics of malignant squamous differentiation. There are also atypical squamous cells with pleomorphic nuclei and high nuclear-to-cytoplasmic ratios, which support the diagnosis of squamous cell carcinoma originating from the teratoma. The histologic features illustrate the reality of malignant transformation of the teratomatous elements.

Histopathological evaluation confirmed the diagnosis of an invasive SCC arising in a mature cystic teratoma. H&E-stained sections displayed sheets of atypical squamous epithelial cells with superficial hornification and intercellular bridges, which are typical of SCC (Figure 3). Tissue samples were obtained from the surgical resection of the ovarian mass, fixed in 10% neutral-buffered formalin (NBF), embedded in paraffin, sectioned at 4 microns, and stained with H&E according to standard procedures in pathology [9]. All procedures performed for the study were approved and conducted in accordance with institutional ethical guidelines and approved protocols.

The immunohistochemical stains were positive for p63, CK 5/6, and p16, but the degree of staining and distribution of expression varied between cases, further contributing to squamous lineage potential. Representative tissue samples were sectioned at 4 μm thickness, fixed in 10% NBF, and embedded in paraffin. Immunohistochemistry (IHC) was performed for each marker simultaneously, but as a separate IHC imaging study on an auto-stainer, i.e., the Ventana BenchMark (Ventana Medical Systems, Inc., Tucson/Oro Valley, AZ, USA). Where appropriate, chromogenic detection was used for each marker - i.e., p63 and CK 5/6 - resulting in squamous differentiation, and p16, indicating possible HPV-mediated oncogenic transformation. The use of IHC studies was necessary to confirm H&E observations, but also to exclude mimickers of squamous carcinoma within the teratomatous stroma. Immunofluorescence was not performed. All biomarkers were assessed on separate serial sections of the pathologic specimen. Other observations included a thyroid follicular neoplasm in the teratomatous elements and invasive carcinoma in the mesoappendix (negative margins were annotated). A total of 19 lymph nodes were recovered, and they were negative for evidence of metastatic disease. Final staging was FIGO (International Federation of Gynecology and Obstetrics) Stage IIIB due to involvement of the peritoneum and intraoperative tumor rupture.

Surgery

She underwent cytoreductive surgery, including diagnostic laparoscopy, exploratory laparotomy, radical pelvic dissection with bilateral salpingo-oophorectomy, supracervical hysterectomy, omentectomy, appendectomy, and cystoscopy.

Postoperative days 1-3

The patient had an uncomplicated postoperative course. She was ambulated on postoperative day 1, tolerated oral intake on postoperative day 2, and was discharged on postoperative day 3 in stable condition. After discharge, she had mild nausea and constipation, which resolved with supportive care and dietary modification.

Postoperative week 1

During her outpatient follow-up, pathology findings, prognosis, and treatment options were discussed with her and her family in detail. The case was reviewed at the institutional multidisciplinary tumor board. Due to the aggressive histology, intraoperative spillage, and advanced stage, the treatment team recommended adjuvant chemotherapy with a platinum-based regimen (carboplatin and paclitaxel), which would be initiated within three weeks of her surgery, at the discretion of the attending consultant. Genetic counseling was also discussed due to the uncommon histology and age at presentation, with germline testing (TP53 and BRCA1/2) pending.

Given her age, medical history (including hypertension, hypothyroidism, and osteoarthritis), and her limited support at home, the surgical and adjuvant plans were carefully considered. In light of her functional independence and social circumstances, these factors were incorporated into her early postoperative ambulation, appropriate discharge planning, and scheduling for chemotherapy. 

**Figure 4:**
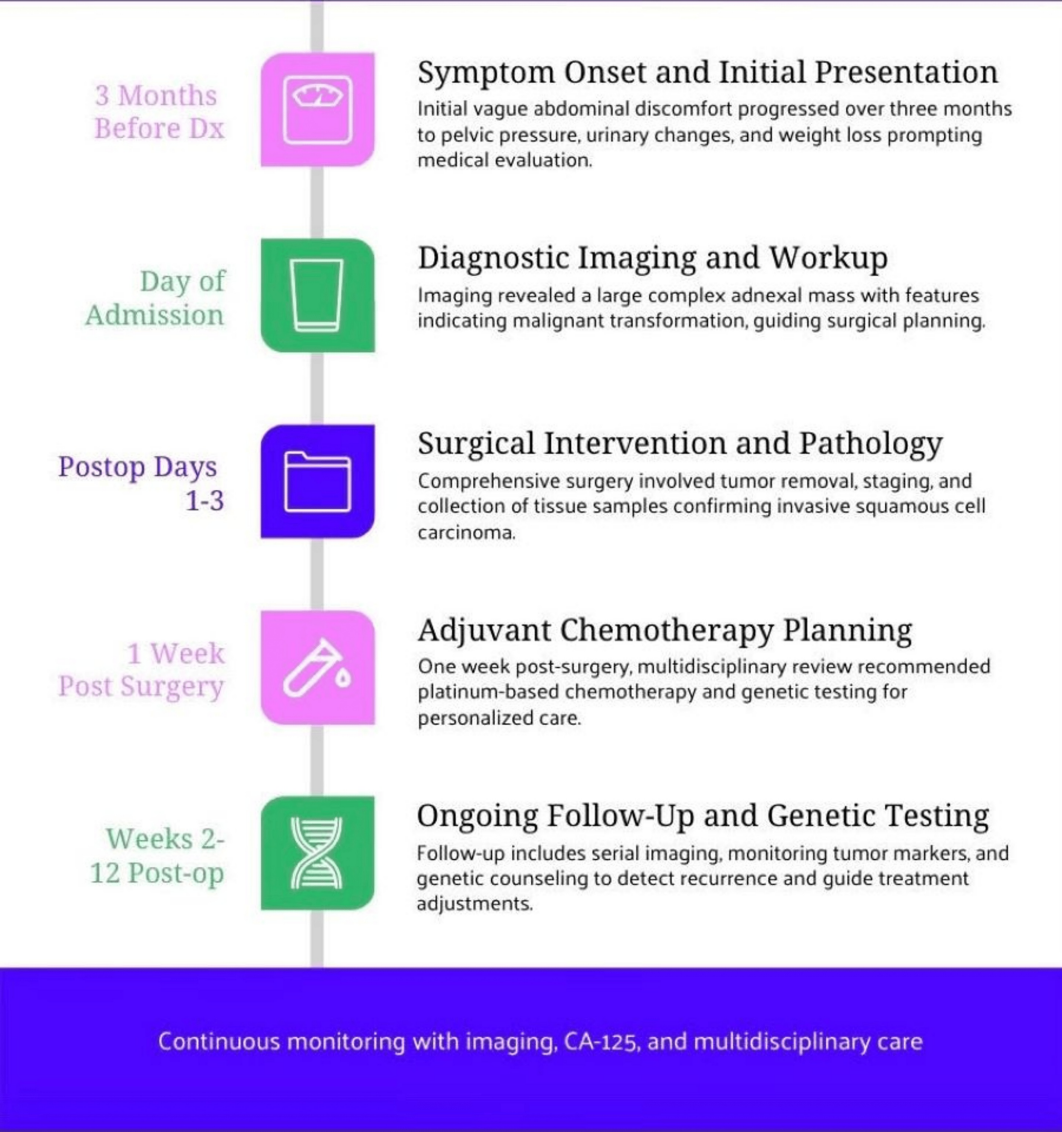
Clinical timeline of the patient A clinical timeline for a 74-year-old woman diagnosed with Stage IIIB ovarian squamous cell carcinoma, originating in a mature teratoma, is presented. Significant events include the onset of symptoms, imaging, surgery, pathology confirmation of the diagnosis, initiation of adjuvant chemotherapy, and follow-up. The timeline demonstrates a coherent and multidisciplinary approach and includes significant treatment milestones.

A detailed summary of the patient’s clinical course, from symptom onset through treatment and follow-up, is illustrated in Figure 4.

A structured summary of the patient's essential clinical, radiologic, pathologic, and therapeutic observations is provided in Table 1. This includes the size and ''extension'' of the ''adnexal'' mass, appendiceal involvement, the histologic diagnosis of SCC arising in a mature teratoma, and factors important for T-staging and treatment. The relevance of significant tumor spillage at the time of the procedure, and mesoappendiceal involvement for determining the FIGO Stage IIIB - and ultimately recommending more aggressive surgical cytoreduction followed by platinum-based adjuvant chemotherapy - was meaningful. While the table is presented as a ''structured'' summary, it serves as a brief reference to highlight the complexity and high-risk nature of this unique malignancy.

**Table 1:** Summary of key clinical, radiologic, pathologic, and therapeutic features of the patient This table provides an at-a-glance overview relevant to diagnosis, staging, and treatment decisions.

Finding	Details
Pelvic Mass Size	27 x 13 x 8.5 cm
Ovarian Involvement	Bilateral salpingo-oophorectomy required
Appendiceal Involvement	Present (mesoappendiceal carcinoma)
Squamous Cell Carcinoma	Malignant transformation from teratoma
Intraoperative Spillage	Positive for malignant cells
Stage	IIIB (due to appendix involvement)

## Discussion

Ovarian cancer and malignant transformation of teratomas

Mature teratomas are generally benign tumors that arise from germ cells and can contain a mix of different tissues, including those from the ectoderm, mesoderm, and endoderm. However, malignant transformation of these teratomas, particularly into SCC, is a rare occurrence, making up about 1%-2% of ovarian cancers. While the exact reasons behind this transformation are still not fully understood, it’s thought to be linked to factors like the presence of mature tissue, hormonal influences, and various genetic elements [10].

The malignant transformation here occurred at an unusually advanced age, which raises the question of risk modifiers. Although there is insufficient data to speculate, some possibilities are chronic inflammation related to the long-standing teratoma, age-related genomic instability, and immune senescence, predisposing to a late transformation to malignant disease.

In this particular case, the patient presented with Stage IIIB ovarian cancer, which involved the mesoappendix and showed signs of tumor spillage during surgery, indicating that the disease was quite advanced.

Mesoappendiceal involvement is relatively rare but signifies tumor extension to the peritoneal structures, which is a very strong predictor of poor prognosis and directly contributes to FIGO IIIB staging. This type of anatomical spread greatly enhances the likelihood of microscopic residual disease, despite surgical margins being cleared, and also underscores the need for adjunct systemic therapy. Furthermore, tumor rupture or dissemination intra-operatively is associated with a higher incidence of peritoneal recurrence and lower progression-free survival (PFS), and a combination of these two findings would, therefore, be particularly high risk [11].

The detection of SCC underscores the tumor's aggressive nature, highlighting the need for thorough surgical intervention and careful postoperative monitoring.

Surgical approach and management

The surgical plan for this patient included a bilateral salpingo-oophorectomy, supracervical hysterectomy, omentectomy, appendectomy, and cystoscopy, all of which align with the standard treatment protocols for advanced-stage ovarian cancer, especially when malignant transformation is involved. Rather than a routine approach, the decision-making in this case was tailored to maximize tumor clearance, given the intraoperative evidence of spillage and mesoappendiceal extension. The extensive nature of the surgery is crucial for effective tumor debulking and reducing any remaining tumor mass. The intraoperative findings of tumor spillage and the presence of malignant cells in the pelvic washings are alarming and further stress the importance of adjuvant chemotherapy to tackle any potential micrometastasis [12].

Postoperative care

Fortunately, the patient’s recovery went smoothly, and she was able to go home on the second day after surgery, having met all her recovery goals. Initially, she experienced some mild gastrointestinal issues, like vomiting, but these were managed effectively with dietary changes. During her follow-up, discussions about the necessity of adjuvant chemotherapy took place, as her overall health was good, making her a strong candidate for additional treatment.

Adjuvant chemotherapy and prognosis

When it comes to advanced-stage ovarian cancer, adjuvant chemotherapy using platinum-based regimens is the go-to treatment, and that’s exactly what’s recommended here. The patient’s situation will be reviewed at a tumor board, where experts will weigh in on the best treatment options, considering the unique details of her pathology, particularly the SCC that has developed from a mature teratoma [13].

While histology differs, molecular data in epithelial ovarian cancers present some relevant parallels. For example, in high-grade serous ovarian carcinoma, BRCA mutations and TP53 alterations are powerful prognostic and therapeutic determinants, especially when considering drugs such as PARP inhibitors. Although this area has not been as extensively studied with SCC transformation, identifying these alterations could provide new avenues for therapeutic agents or clinical trials in the future. Hence, the pending BRCA/TP53 results are likely to have prognostic and potential therapeutic relevance in this rare case [14].

**Figure 5:**
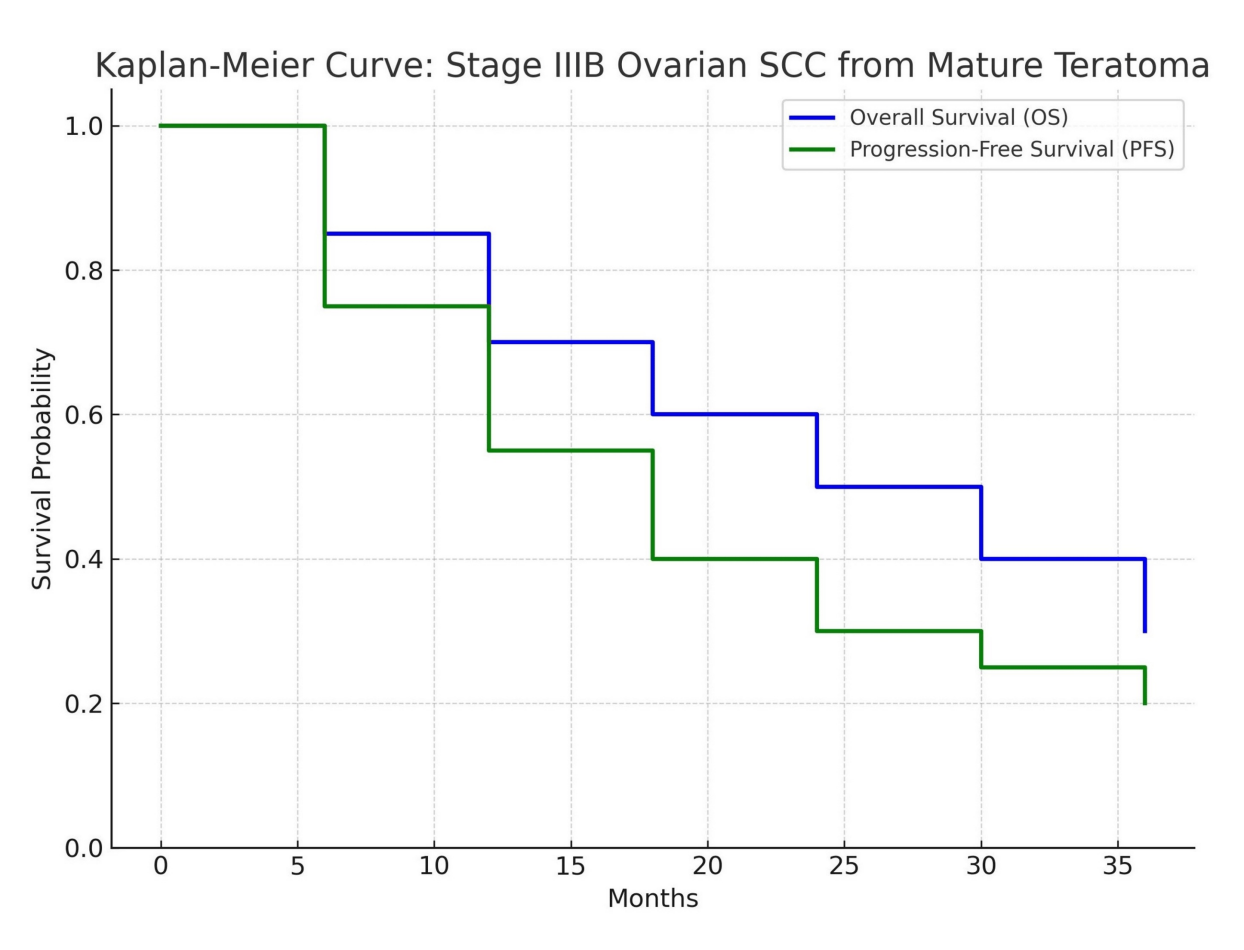
Kaplan-Meier survival curves Kaplan-Meier survival curves are depicted, demonstrating progression-free survival (PFS) and overall survival (OS) for patients with FIGO (International Federation of Gynecology and Obstetrics) Stage IIIB ovarian cancer from the literature (n = 112, SEER database, 2013-2018). The figure helps establish the expected prognosis, which adds utility in discussing the patient’s outcome in the current case. Figure constructed by the authors, based on pooled survival data from recent case series [7,15]. SCC, squamous cell carcinoma

Kaplan-Meier survival analyses from recent case series show that patients with advanced-stage ovarian SCC arising from mature teratoma have dramatically inferior PFS and overall survival (OS) when compared to patients with standard epithelial ovarian cancer. Median PFS for Stage IIIB is reported as 12 to 18 months, with a two-year OS of less than 50%, all indicating the aggressive nature of this rare transformation (Figure 5). For instance, Tokunaga et al. reported two-year OS rates of approximately 30% for Stage III disease and 0% for Stage IV, with median PFS in Stage III ranging from 12 to 18 months [15]. Although informative, the Kaplan-Meier results arise from pooled case series, so penetration of the cases is very rare; therefore, the survival estimates should be approached with caution in terms of generalizability. However, these estimates are useful to underscore the poor prognosis and necessity for systemic treatment early in the course in this population. These data reaffirm the importance of early initiation of platinum-based chemotherapy and stringent clinical monitoring to optimize outcomes and potentially improve survival in patients with aggressive or advanced-stage disease.

Follow-up surveillance

In these circumstances, follow-up would be individualized but typically would consist of cross-sectional imaging, such as CT or MRI, every three to six months for the first two years, then at increasing intervals thereafter. CA-125 is a common surveillance marker for epithelial ovarian cancers; its role, however, in SCC from teratomas is not established. Nevertheless, it may still be useful to trend with imaging. PET-CT may be considered if equivocal findings are observed or to assess for recurrence. As there are no consensus guidelines for follow-up or surveillance, consultation with multidisciplinary input and considerations for individualized planning are crucial [16].

Genetic testing and surveillance

Although the pathological findings indicate a malignant transformation rather than a primary spontaneous ovarian cancer, genetic testing is a crucial step. It helps identify any hereditary factors that could influence the patient's treatment plan and future monitoring [17]. During surveillance visits, the focus will be on keeping an eye out for any signs of recurrence and managing the side effects that come with chemotherapy [18].

Management of advanced ovarian cancer

Effectively managing advanced ovarian cancer requires a combination of surgical intervention and aggressive chemotherapy, along with vigilant monitoring for any recurrence. As highlighted in various studies, having a multidisciplinary team is essential for achieving the best outcomes, especially for patients facing rare transformations like SCC from mature teratomas. In this particular case, the integration of surgery, adjuvant therapy, and planned genetic evaluation underscores a personalized and aggressive treatment plan aimed at maximizing survival. Thanks to advancements in surgical techniques and new chemotherapy drugs, patient prognosis and survival rates are on the rise, though treating older patients with other health issues still presents significant challenges [19].

## Conclusions

The case illustrates the difficulties in managing rare, high-grade ovarian tumors, including SCC, which is derived from mature teratoma. In a select population of elderly patients with good performance status, aggressive surgical resection and adjuvant chemotherapy are appropriate and effective, and serve as a reminder that this initial curative intention should not be limited solely based on age. The treatment course was structured through a multidisciplinary approach and shared decision-making. This case is an important example of individualized oncologic care and demonstrates how personalized care can result in favorable outcomes, even in challenging, high-risk cases. Prompt imaging and referral may be indicated by early clinical signs, such as nonspecific abdominal discomfort, postmenopausal pelvic masses, or an unexplained rise in inflammatory markers (e.g., CRP and leukocytosis). Recognition of these early signs is important for timely diagnosis and treatment in high-risk cases. Continued surveillance and consideration of genetic work-up are important aspects of long-term follow-up.
